# Relationship between triglyceride-glucose index and aminotransferase among Royal Thai Army personnel 2017–2021: a serial cross-sectional study

**DOI:** 10.1186/s12944-023-01811-5

**Published:** 2023-04-03

**Authors:** Sethapong Lertsakulbunlue, Mathirut Mungthin, Ram Rangsin, Anupong Kantiwong, Boonsub Sakboonyarat

**Affiliations:** 1 Department of Pharmacology, Phramongkutklao College of Medicine, Bangkok, 10400 Thailand; 2 Department of Parasitology, Phramongkutklao College of Medicine, Bangkok, 10400 Thailand; 3 Department of Military and Community Medicine, Phramongkutklao College of Medicine, Bangkok, 10400 Thailand

**Keywords:** Triglyceride glucose index, Prevalence, Association, Relationship, Aminotransferase, NAFLD, Thailand

## Abstract

**Background:**

Insulin resistance (IR) is a major pathogenesis of nonalcoholic fatty liver disease (NAFLD). The triglyceride-glucose (TyG) index has recently gained popularity to assess IR and NAFLD due to its simplicity and low cost. The aim of the current study was to evaluate the relationship between the TyG index and aminotransferase.

**Methods:**

A serial cross-sectional study was conducted among 232,235 Royal Thai Army (RTA) personnel aged 35–60 years from 2017–2021. Elevated aminotransferase was defined as ≥ 40 U/L and ≥ 35 U/L among males and females, respectively. A linear regression analysis between the TyG index and log-transformed aminotransferase was performed. High- and low-TyG index groups were divided according to Youden’s index cut point for predicting elevated aminotransferase. Multivariable logistic analysis was also utilized to investigate the association between the TyG index and elevated aminotransferase.

**Results:**

The TyG index revealed a dose‒response relationship with log-transformed aminotransferase in both sexes and all age groups. The TyG index was positively associated with the prevalence of elevated aminotransferases. In comparison with the first TyG quartile (< 8.37), participants in the fourth quartile (> 9.23) had a higher chance for elevated ALT (AOR: 2.81, 95% CI: 2.71–2.90 for males and AOR: 4.01, 95% CI: 3.50–4.60 for females, *P* < 0.001 for both). In the fourth TyG quartile, the prevalence of elevated ALT was 47.8% and 40.2% in the participants aged 35–44 and male participants, respectively.

**Conclusion:**

A high TyG index is a novel risk factor for elevated aminotransferase among RTA personnel. Those with a high TyG index should be screened for elevated aminotransferase, particularly males aged 35–44 years.

**Supplementary Information:**

The online version contains supplementary material available at 10.1186/s12944-023-01811-5.

## Background

Nonalcoholic fatty liver disease (NAFLD) is one of the most common causes of chronic liver disease, resulting in cirrhosis and hepatocellular cancer [1]. In 2020, the prevalence of NAFLD in Asia reached 29.6%, surpassing that of Western populations [2]. Currently, liver biopsy is the gold standard for diagnosis, as it is invasive and expensive [3]. Imaging, such as ultrasound, might assist in the diagnosis. However, in the early stage, ultrasonography might reveal normal findings. Aminotransferase and bilirubin levels are elevated in hepatocellular injury and may aid in the early detection of NAFLD [4]. NAFLD is the most common cause of asymptomatic aminotransferase elevation [5, 6]. Despite testing aminotransferases among Royal Thai Army (RTA) personnel, only those above 35 years old were tested. Furthermore, measuring aminotransferase levels is not mandatory for health screening in Thailand [7].

Insulin resistance (IR) is known to be the primary mechanism of NAFLD [8]. However, no specific method has been established to determine IR. Currently, the gold standard tests are the euglycemic insulin clamp and the intravenous glucose tolerance test, both of which are invasive and expensive. Consequently, they are rarely utilized in clinical practice. Nevertheless, in 2008, Simental-Mendia et al. proposed the triglyceride-glucose (TyG) index as a surrogate of IR, and Guerrero-Romero et al. confirmed the hypothesis in 2010 [9]. Guerrero-Romero et al. also suggested that the TyG index may reflect hepatic insulin resistance due to its association with liver fat composition [10]. In addition, a meta-analysis in 2022 demonstrated that a high TyG index was positively associated with NAFLD pooled OR (6.00, CI: 4.12–8.74) and pooled HR (1.70, CI: 1.28–2.27) in all nationalities, regardless of whether they had diabetes [3].

Recently, Sakboonyarat et al. revealed that metabolic syndrome was a common health issue among RTA personnel over 35 years old. Moreover, rising trends in obesity prevalence were also observed in the population [11, 12]. Some related studies indicated an association between the TyG index and aminotransferase in a healthy obese population [13, 14]. However, in Asia, more than one-fifth of those with NAFLD are not obese [15]. Furthermore, the association between the TyG index and elevated aminotransferase has yet to be reported in Thailand. Therefore, the present study aimed to investigate the relationship between the TyG index and aminotransferase and the ability of the TyG index to predict elevated aminotransferase among RTA personnel through the use of the RTA personnel database of annual health examinations during 2017–2021 provided by the RTA Medical Department (RTAMED).

## Methods

### Study design and subjects

**Fig. 1:**
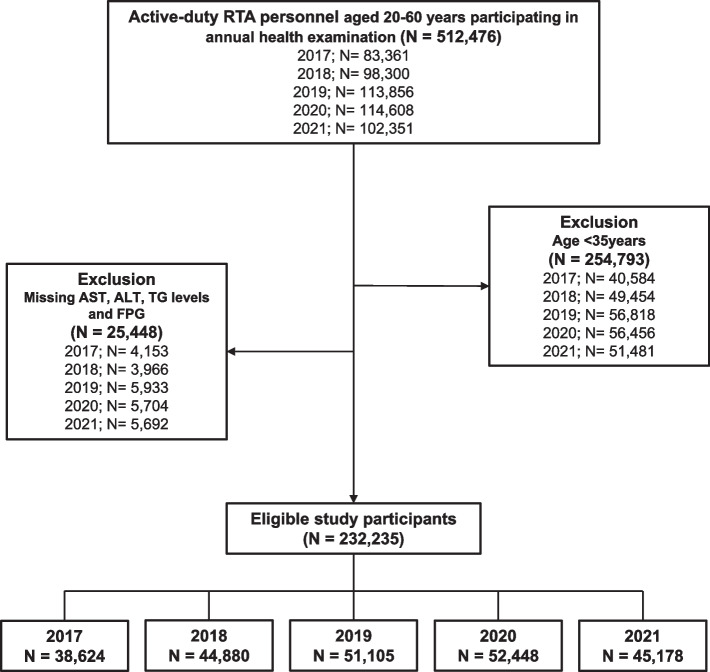
Flowchart of the enrolled participants

From 2017 to 2021, a serial cross-sectional study was carried out. The data set was extracted from the RTA personnel annual health examination database with permission from RTAMED in Bangkok, Thailand [16]. Annually, the RTAMED provides health examinations for RTA personnel through the Army Institute of Pathology, the Armed Forces Research Institute of Medical Sciences, Phramongkutklao Hospital in Bangkok, and 36 RTA hospitals nationwide. The results of the health examinations were reported to the RTAMED in Bangkok. A total of 512,476 active-duty RTA personnel aged 20–60 years were enrolled in the current study. Those under 35 years (254,793 RTA personnel) were excluded due to the absence of an aminotransferase test. In addition, 25,448 subjects were excluded from the study because their laboratory tests for aspartate aminotransferase (AST), alanine aminotransferase (ALT), fasting plasma glucose (FPG), and triglyceride (TG) were incomplete. Finally, the present study included 232,235 RTA personnel from 2017 to 2021 (Fig. 1).

### Data collection

The Army Institute of Pathology, the Armed Forces Research Institute of Medical Sciences, and 37 RTA hospitals conduct annual health examinations for RTA personnel. A self-report guide was utilized to collect demographic characteristics and behavioral risk factors, including age, sex, health insurance schemes, regular exercise, alcohol use, and smoking status. Anthropometric measurements of systolic blood pressure (SBP) and diastolic blood pressure (DBP) were taken. Moreover, bodyweight and height were measured to calculate body mass index (BMI).

Cardiometabolic risk factors include high FPG, SBP, DBP, and serum uric levels. FPG was categorized as under 126 mg/dL and 126 mg/dL or more (hyperglycemia). High blood pressure was defined as an SBP of 140 mmHg or more or a DBP of 90 mmHg or more. High serum uric level was defined as 7 mg/dL or more [17, 18]. Elevated aminotransferase was defined as AST or ALT equal to or greater than 40 U/L and 35 U/L among males and females, respectively [19]. The equation ln [TG (mg/dL)*FBG (mg/dL)/2] was utilized to calculate the TyG index [9]. The high and low TyG indexes were divided by the cutoff point using Youden’s J statistics of the ROC curve of the TyG index in predicting elevated AST and ALT.

### Statistical analyses

The frequency distribution of demographic characteristics was performed to describe the study subjects. Categorical data are presented as percentages. Regarding continuous variables, the mean and standard deviation (SD) are presented in the case of a normal distribution, and the median and interquartile range (IQR) are presented in the case of a nonnormal distribution. Log transformation was performed on AST and ALT due to their nonnormal distribution, and the linear assumption was violated. The Breusch–Pagan test was utilized to assess homoscedasticity. Linear regression analysis was used to identify the linear association between the TyG index and log-transformed AST and ALT. Youden’s J statistics of the ROC curve were utilized to determine the TyG cutoff point in predicting elevated AST and ALT. Then, an area under the ROC curve (AUC) was calculated. Logistic regression analysis was used to determine the association between elevated aminotransferase and the TyG index. Furthermore, multivariable analysis was employed to estimate the adjusted odds ratio (OR) and 95% confidence interval (CI). All statistical tests were two-sided, and a *P* value less than 0.05 was considered statistically significant. All analyses were performed using StataCorp, 2021, *Stata Statistical Software: Release 17*. College Station, TX: StataCorp LLC.

## Results

### Baseline characteristics of participants

Table 1 presents the demographic, behavioral, and laboratory characteristics of the participants. Approximately 90% of the participants were males. The mean age of the participants ranged from 46.7 to 48.0 years. Over half of the participants resided in the central region. A quarter of the study had high BP, and a tenth had hyperglycemia. The median fasting TG level decreased from 136 mg/dL to 130 mg/dL in 2017–2020 and increased to 131 mg/dL in 2021. The average TyG index ranged between 8.9 and 9.0 in 2017–2019, decreased to 8.8 in 2020, and increased slightly in 2021. The median AST and ALT were in the ranges of 24–25 and 25–28, respectively. The prevalence of elevated ALT was 28.2, 27.0, 25.2, 22.1, and 24.8% in 2017–2021, respectively, and approximately 13% had elevated AST.

**Table 1:** Demographic, behavioral characteristics and laboratory tests of participants from 2017 to 2021 (*N* = 232,235)

Year	2017	2018	2019	2020	2021
**Characteristics**	**n (%)**	**n (%)**	**n (%)**	**n (%)**	**n (%)**
**No. of participants**	38,624	44,880	51,105	52,448	45,178
**Sex**					
Male	35,064 (90.78)	39,879 (88.86)	45,753 (89.53)	46,069 (87.84)	40,723 (90.14)
Female	3560 (9.22)	5001 (11.14)	5352 (10.47)	6379 (12.16)	4455 (9.86)
**Age (years)**					
35–39	6552 (16.96)	8402 (18.72)	10,317 (20.19)	11,275 (21.50)	11,237 (24.87)
40–44	7115 (18.42)	8991 (20.03)	9971 (19.51)	9392 (17.91)	8226 (18.21)
45–49	6124 (15.86)	7122 (15.87)	7892 (15.44)	8632 (16.46)	7865 (17.41)
50–54	9629 (24.93)	9850 (21.95)	10,436 (20.42)	9479 (18.07)	7517 (16.64)
55–60	9204 (23.83)	10,515 (23.43)	12,489 (24.44)	13,670 (26.06)	10,333 (22.87)
Mean ± SD	48.01 ± 7.11	47.57 ± 7.33	47.44 ± 7.50	47.47 ± 7.74	46.74 ± 7.72
**Regions**					
Central	22,403 (58.00)	27,108 (60.40)	29,983 (58.67)	30,421 (58.00)	23,869 (52.83)
Northeast	7004 (18.13)	7219 (16.09)	8503 (16.64)	9945 (18.96)	7611 (16.85)
North	7408 (19.18)	5383 (11.99)	7386 (14.45)	6721 (12.81)	8660 (19.17)
South	1809 (4.68)	5170 (11.52)	5233 (10.24)	5361 (10.22)	5038 (11.15)
**Health Scheme**					
Civil servant medical benefits	37,708 (97.63)	43,998 (98.03)	50,192 (98.21)	50,914 (97.08)	44,425 (98.33)
Social Security	570 (1.48)	423 (0.94)	487 (0.95)	1121 (2.14)	644 (1.43)
Universal Coverage	346 (1.00)	459 (1.02)	426 (0.83)	413 (0.79)	109 (0.24)
**Current smoking**					
No	28,807 (75.61)	32,465 (73.28)	36,518 (73.35)	35,688 (71.39)	32,360 (71.76)
Yes	9294 (24.39)	11,840 (26.72)	13,268 (26.65)	14,301 (28.61)	12,734 (28.24)
**Current alcohol drinking**					
No	13,700 (35.89)	16,481 (37.13)	18,167 (35.70)	16,610 (33.23)	16,578 (36.78)
Yes	24,471 (64.11)	27,909 (62.87)	32,724 (64.30)	33,376 (66.77)	28,490 (63.22)
**Regular exercise**					
No	16,417 (43.17)	18,502 (42.56)	18,540 (37.25)	23,227 (44.87)	20,076 (44.61)
Yes	21,614 (56.83)	24,975 (57.44)	31,228 (62.75)	28,536 (55.13)	24,929 (55.39)
**Body mass index (kg/m**^**2**^**)**					
Mean ± SD	25.12 ± 3.63	25.22 ± 3.66	25.27 ± 3.70	25.28 ± 3.74	25.35 ± 3.78
**Systolic blood pressure (mmHg)**					
Mean ± SD	130.87 ± 16.79	130.91 ± 16.84	131.32 ± 16.69	131.34 ± 16.63	132.16 ± 17.19
**Diastolic blood pressure (mmHg)**					
Mean ± SD	81.50 ± 11.63	81.33 ± 11.66	81.08 ± 11.61	80.84 ± 11.58	81.27 ± 11.88
**Uric acid(mg/dL)**					
Median (IQR)	6.42 ± 1.53	6.26 ± 1.54	6.34 ± 1.53	6.24 ± 1.53	6.43 ± 1.56
**Fasting plasma glucose (mg/dL)**					
< 126	34,714 (89.88)	40,375 (89.96)	45,387 (88.81)	47,314 (90.21)	40,924 (90.58)
≥ 126	3910 (10.12)	4505 (10.04)	5718 (11.19)	5134 (9.79)	4254 (9.42)
Median (IQR)	95 (88–106)	96 (88–106)	95 (88–106)	95 (88–105)	95 (88–105)
**Fasting triglyceride (mg/dL)**					
Median (IQR)	136 (94–202)	135 (94–201)	133 (93–197)	130 (90–191)	131 (91–194)
**Triglyceride-glucose index**					
Quartile 1 (< 8.37)	9268 (24.00)	10,830 (24.13)	12,445 (24.35)	13,827 (26.36)	11,737 (25.98)
Quartile 2 (8.37–8.78)	9490 (24.57)	10,997 (24.50)	12,900 (25.24)	13,212 (25.19)	11,452 (25.35)
Quartile 3 (8.79–9.23)	9915 (25.67)	11,305 (25.19)	12,590 (24.64)	13,012 (24.81)	11,202 (24.80)
Quartile 4 (> 9.23)	9951 (25.76)	11,748 (26.18)	13,170 (25.77)	12,397 (23.64)	10,787 (23.88)
Mean ± SD	8.86 ± 0.68	8.87 ± 0.68	8.86 ± 0.68	8.82 ± 0.66	8.83 ± 0.67
**Aspartate aminotransferase (U/L)**					
< 40 in male or < 35 in female	33,083 (85.65)	39,061 (87.03)	44,725 (87.52)	45,411 (86.58)	39,513 (87.46)
≥ 40 in male or ≥ 35 in female	5541 (14.35)	5819 (12.97)	6380 (12.48)	7037 (13.42)	5665 (12.54)
Median (IQR)	25 (20–32)	25 (20–32)	24 (20–31)	24 (20–31)	25 (20–31)
**Alanine aminotransferase (U/L)**					
< 40 in male or < 35 in female	27,726 (71.78)	32,753 (72.98)	38,217 (74.78)	40,837 (77.86)	33,976 (75.20)
≥ 40 in male or ≥ 35 in female	10,898 (28.22)	12,127 (27.02)	12,888 (25.22)	11,611 (22.14)	11,202 (24.80)
Median (IQR)	28 (19–42)	27 (19–41)	26 (18–39)	25 (18–37)	26 (18–39)

*SD* standard deviation, *IQR* interquartile range

#### Linear regression analysis for the relationship between the triglyceride-glucose index and aminotransferase

**Fig. 2:**
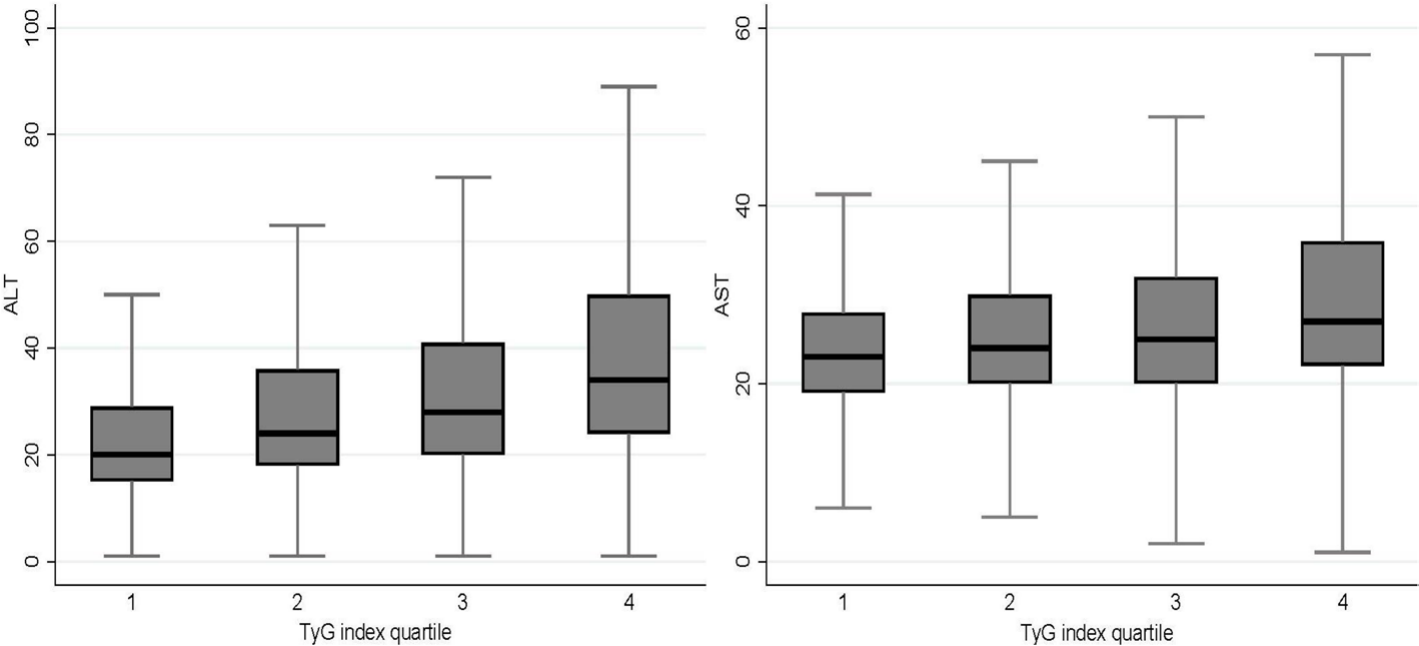
Boxplot of aminotransferases stratified by triglyceride-glucose (TyG) index quartiles

The median for ALT was 20, 24, 28, and 34 for TyG index quartiles 1 to 4, respectively. The median AST increased from 23 to 27 for the first and fourth TyG index quartiles (Fig. 2). Table 2 presents the overall linear regression analysis of log-transformed AST, ALT, and TyG index. After adjusting for confounders including age, sex, BMI, region, scheme, year, smoking, alcohol drinking, exercise, SBP, DBP, and serum uric levels, we observed a higher overall dose‒response relationship between the TyG index and ALT (adjusted β = 0.17, 95% CI: 0.17–0.17, *P* < 0.001) in comparison with AST (adjusted β = 0.07, 95% CI: 0.07–0.07, *P* < 0.001). Finally, Table 3 illustrates the sex- and age-specific multivariable linear regression analysis of log-transformed AST, ALT, and TyG index. Females had a stronger dose‒response relationship between the TyG index and ALT than males (adjusted β = 0.18, 95% CI: 0.17–0.19 for females and adjusted β = 0.16, 95% CI: 0.16–0.17 for males). According to age groups, the adjusted β coefficient between the TyG index and the log-transformed ALT was lower in higher age groups, at 0.19, 0.16, and 0.14 at ages 35–44, 45–54, and ≥ 55, respectively. Meanwhile, the adjusted β coefficient between the TyG index and the log-transformed AST was 0.09 and 0.05 at ages 35–44 and ≥ 55, respectively.

**Table 2:** Linear regression analysis of log-transformed AST, ALT and Triglyceride-glucose (TyG) index

**Variables**	**Univariable**			**Multivariable**^a^			
	**β Coefficient**	**95% CI**	***p* value**	**β Coefficient**		**95% CI**	***p* value**
**Log-transformed aspartate aminotransferase (AST)**							
**TyG index**	0.12	0.12–0.13	< 0.001	0.07	0.07–0.07		< 0.001
**TyG index (Quartiles)**							
Quartile 1 (< 8.37)	ref			ref			
Quartile 2 (8.37–8.78)	0.06	0.05–0.06	< 0.001	0.01	0.01–0.02		< 0.001
Quartile 3 (8.79–9.23)	0.11	0.10–0.11	< 0.001	0.04	0.03–0.04		< 0.001
Quartile 4 (> 9.23)	0.22	0.21–0.22	< 0.001	0.12	0.11–0.12		< 0.001
**Log-transformed alanine aminotransferase (ALT)**							
**TyG index**	0.27	0.27–0.27	< 0.001	0.17	0.17–0.17		< 0.001
**TyG index (Quartiles)**							
Quartile 1 (< 8.37)	ref			ref			
Quartile 2 (8.37–8.78)	0.18	0.17–0.19	< 0.001	0.09	0.09–0.10		< 0.001
Quartile 3 (8.79–9.23)	0.32	0.31–0.32	< 0.001	0.18	0.17–0.19		< 0.001
Quartile 4 (> 9.23)	0.49	0.48–0.50	< 0.001	0.30	0.30–0.31		< 0.001

^a^Adjusted for age, sex, body mass index, region, scheme, year, smoking status, alcohol use, exercise, systolic blood pressure, diastolic blood pressure, serum uric acid

**Table 3:** Sex- and Age-specific multivariable linear regression analysis of log-transformed AST, ALT and Triglyceride-glucose (TyG) index

Variables	Log-transformed aspartate aminotransferase (AST)			Log-transformed alanine aminotransferase (ALT)		
	β coefficient	**95% CI**	***p* value**	β coefficient	**95% CI**	***p* value**
**Male**^a^						
**TyG index**	0.07	0.07–0.08	< 0.001	0.16	0.16–0.17	< 0.001
**TyG index (Quartiles)**						
Quartile 1 (< 8.37)	ref			ref		
Quartile 2 (8.37–8.78)	0.01	0.01–0.02	< 0.001	0.09	0.08–0.10	< 0.001
Quartile 3 (8.79–9.23)	0.04	0.03–0.04	< 0.001	0.18	0.17–0.18	< 0.001
Quartile 4 (> 9.23)	0.12	0.11–0.12	< 0.001	0.30	0.29–0.30	< 0.001
**Female**^a^						
**TyG index**	0.06	0.05–0.07	< 0.001	0.18	0.17–0.19	< 0.001
**TyG index (Quartiles)**						
Quartile 1 (< 8.37)	ref			ref		
Quartile 2 (8.37–8.78)	0.02	0.01–0.03	0.002	0.09	0.07–0.10	< 0.001
Quartile 3 (8.79–9.23)	0.05	0.04–0.06	< 0.001	0.18	0.16–0.20	< 0.001
Quartile 4 (> 9.23)	0.13	0.12–0.15	< 0.001	0.33	0.31–0.36	< 0.001
**Age 35-44**^b^						
**TyG index**	0.09	0.08–0.09	< 0.001	0.19	0.18–0.20	< 0.001
**TyG index (Quartiles)**						
Quartile 1 (< 8.37)	ref			ref		
Quartile 2 (8.37–8.78)	0.02	0.01–0.03	< 0.001	0.11	0.09–0.12	< 0.001
Quartile 3 (8.79–9.23)	0.06	0.05–0.06	< 0.001	0.21	0.19–0.22	< 0.001
Quartile 4 (> 9.23)	0.15	0.14–0.16	< 0.001	0.35	0.34–0.36	< 0.001
**Age 45-54**^b^						
**TyG index**	0.08	0.07–0.08	< 0.001	0.16	0.16–0.17	< 0.001
**TyG index (Quartiles)**						
Quartile 1 (< 8.37)	ref			ref		
Quartile 2 (8.37–8.78)	0.02	0.01–0.03	0.001	0.10	0.09–0.11	< 0.001
Quartile 3 (8.79–9.23)	0.05	0.04–0.05	< 0.001	0.19	0.17–0.20	< 0.001
Quartile 4 (> 9.23)	0.12	0.11–0.13	< 0.001	0.30	0.29–0.31	< 0.001
**Age ≥ 55**^b^						
**TyG index**	0.05	0.05–0.06	< 0.001	0.14	0.14–0.15	< 0.001
**TyG index (Quartiles)**						
Quartile 1 (< 8.37)	ref			ref		
Quartile 2 (8.37–8.78)	0.00	(-0.01)-0.01	0.637	0.06	0.05–0.07	< 0.001
Quartile 3 (8.79–9.23)	0.01	0.01–0.02	< 0.001	0.14	0.13–0.15	< 0.001
Quartile 4 (> 9.23)	0.08	0.07–0.09	< 0.001	0.24	0.23–0.26	< 0.001

^a^Adjusted for age, body mass index, region, scheme, year, smoking status, alcohol use, exercise, systolic blood pressure, diastolic blood pressure, serum uric acid

^b^Adjusted for sex, body mass index, region, scheme, year, smoking status, alcohol use, exercise, systolic blood pressure, diastolic blood pressure, serum uric acid

#### Logistic regression analysis for the relationship between triglyceride-glucose index and elevated aminotransferase

**Fig. 3:**
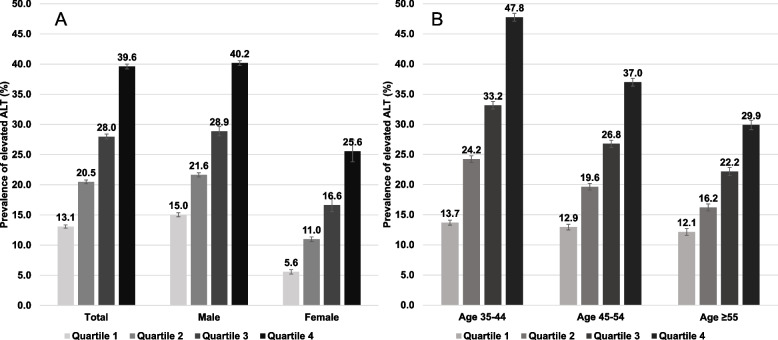
Prevalence of elevated ALT stratified by triglyceride-glucose (TyG) index quartiles and by **(A)** sex and **(B)** age

**Fig. 4:**
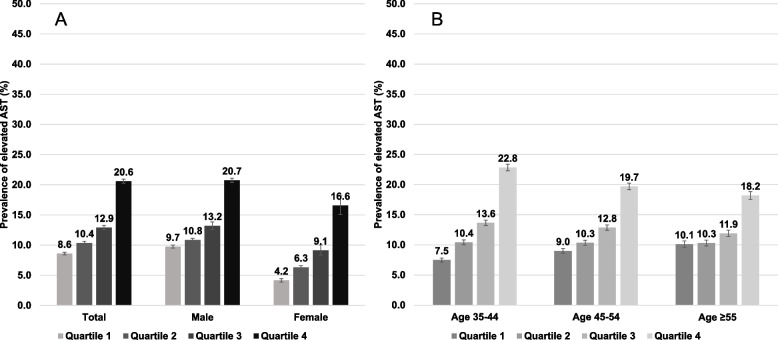
Prevalence of elevated AST stratified by triglyceride-glucose (TyG) index quartiles and by **(A)** sex and **(B)** age

Figure 3 reveals the prevalence of elevated ALT stratified by TyG index quartiles. Figure 3a demonstrates that among males, the prevalence of elevated ALT was 40.2% in the highest quartile and 15.0% in the lowest quartile, whereas among females, the highest and lowest quartiles were 25.6% and 5.6%, respectively. Those in the fourth TyG index quartile, aged 35–44, had the highest prevalence of elevated ALT of 47.8% (Fig. 3b). Figure 4 displays the prevalence of elevated AST stratified by TyG index quartiles. For AST, a higher prevalence of elevated AST was also shown with increasing TyG index quartile for both sexes and all groups.

Table 4 demonstrates the logistic regression analysis of elevated AST, ALT, and TyG index. After adjusting for confounders, a unit increase in the TyG index increased the odds of elevated AST and ALT by 1.56 and 1.77, respectively (*P* < 0.001). A dose‒response relationship was observed between elevated aminotransferases and TyG index quartiles. After using the cut point of the ROC curve analysis to determine the high TyG index (> 8.93 for AST and > 8.84 for ALT) and low TyG index for AST and ALT, a high TyG index was associated with elevated AST and ALT (AOR: 1.60, 95% CI: 1.56–1.65, *P* < 0.001 for AST and AOR: 1.90, 95% CI: 1.86–1.94, *P* < 0.001 for ALT). A high TyG index was also associated with elevated AST and ALT in all sexes and age groups, as shown in Table 5. Furthermore, those in the fourth quartile revealed strong odds of elevated ALT in comparison with the first quartile (AOR: 2.81, 95% CI: 2.71–2.90 for males and AOR: 4.01, 95% CI: 3.50–4.60 for females, *P* < 0.001 for both).

**Table 4:** Logistic regression analysis of elevated AST, ALT and Triglyceride-glucose (TyG) index

Variables	Total					
	**OR**	**95% CI**	***p* value**	**AOR**^a^	**95% CI**	***p* value**
**Elevated aspartate aminotransferase (AST)**						
**TyG index**	1.83	1.80–1.86	< 0.001	1.56	1.54–1.59	< 0.001
**TyG index**						
Low TyG index ≤ 8.93	ref			ref		
High TyG index > 8.93	2.00	1.95–2.05	< 0.001	1.60	1.56–1.65	< 0.001
**TyG index (Quartiles)**						
Quartile 1 (< 8.37)	ref			ref		
Quartile 2 (8.37–8.78)	1.23	1.18–1.28	< 0.001	1.08	1.04–1.13	< 0.001
Quartile 3 (8.79–9.23)	1.58	1.52–1.64	< 0.001	1.27	1.22–1.32	< 0.001
Quartile 4 (> 9.23)	2.76	2.66–2.86	< 0.001	1.98	1.91–2.06	< 0.001
**Elevated alanine aminotransferase (ALT)**						
**TyG index**	2.17	2.14–2.20	< 0.001	1.77	1.74–1.80	< 0.001
**TyG index**						
Low TyG index ≤ 8.84	ref			ref		
High TyG index > 8.84	2.52	2.47–2.57	< 0.001	1.90	1.86–1.94	< 0.001
**TyG index (Quartiles)**						
Quartile 1 (< 8.37)	ref			ref		
Quartile 2 (8.37–8.78)	1.71	1.66–1.77	< 0.001	1.43	1.38–1.48	< 0.001
Quartile 3 (8.79–9.23)	2.58	2.50–2.66	< 0.001	1.91	1.85–1.98	< 0.001
Quartile 4 (> 9.23)	4.36	4.23–4.49	< 0.001	2.88	2.79–2.98	< 0.001

*OR* odds ratio, *AOR* adjusted odds ratio

^a^Adjusted for age, sex, body mass index, region, scheme, year, smoking status, alcohol use, exercise, systolic blood pressure, diastolic blood pressure, serum uric acid

**Table 5:** Sex-specific and age-specific multivariable logistic regression analysis of elevated AST, ALT and Triglyceride-glucose (TyG) index

Variables	Elevated aspartate aminotransferase (AST)			Elevated alanine aminotransferase (ALT)		
	**AOR**	**95% CI**	***p* value**	**AOR**	**95% CI**	***p* value**
**Male**^**a**^						
**TyG index**	1.55	1.52–1.58	< 0.001	1.75	1.72–1.78	< 0.001
**TyG index**						
Low TyG index^c^	ref			ref		
High TyG index^c^	1.58	1.54–1.62	< 0.001	1.88	1.84–1.92	< 0.001
**TyG index (Quartiles)**						
Quartile 1 (< 8.37)	ref			ref		
Quartile 2 (8.37–8.78)	1.05	1.00–1.09	0.032	1.40	1.35–1.44	< 0.001
Quartile 3 (8.79–9.23)	1.22	1.17–1.27	< 0.001	1.86	1.80–1.93	< 0.001
Quartile 4 (> 9.23)	1.90	1.83–1.98	< 0.001	2.81	2.71–2.90	< 0.001
**Female**^**a**^						
**TyG index**	1.96	1.79–2.15	< 0.001	2.24	2.08–2.42	< 0.001
**TyG index**						
Low TyG index^d^	ref			ref		
High TyG index^d^	2.11	1.88–2.37	< 0.001	2.28	2.08–2.50	< 0.001
**TyG index (Quartiles)**						
Quartile 1 (< 8.37)	ref			ref		
Quartile 2 (8.37–8.78)	1.30	1.13–1.50	< 0.001	1.69	1.51–1.90	< 0.001
Quartile 3 (8.79–9.23)	1.74	1.50–2.03	< 0.001	2.42	2.14–2.74	< 0.001
Quartile 4 (> 9.23)	3.27	2.79–3.84	< 0.001	4.01	3.50–4.60	< 0.001
**Age 35–44 years**^b^						
**TyG index**	1.66	1.61–1.72	< 0.001	1.91	1.85–1.96	< 0.001
**TyG index**						
Low TyG index^c^	ref			ref		
High TyG index^c^	1.76	1.68–1.85	< 0.001	2.07	2.00–2.14	< 0.001
**TyG index (Quartiles)**						
Quartile 1 (< 8.37)	ref			ref		
Quartile 2 (8.37–8.78)	1.16	1.08–1.25	< 0.001	1.48	1.40–1.57	< 0.001
Quartile 3 (8.79–9.23)	1.44	1.34–1.54	< 0.001	2.05	1.94–2.16	< 0.001
Quartile 4 (> 9.23)	2.31	2.16–2.48	< 0.001	3.28	3.11–3.47	< 0.001
**Age 45–54 years**^b^						
**TyG index**	1.55	1.50–1.60	< 0.001	1.72	1.67–1.77	< 0.001
**TyG index**						
Low TyG index^c^	ref			ref		
High TyG index^c^	1.59	1.52–1.67	< 0.001	1.86	1.79–1.93	< 0.001
**TyG index (Quartiles)**						
Quartile 1 (< 8.37)	ref			ref		
Quartile 2 (8.37–8.78)	1.10	1.03–1.19	0.009	1.39	1.32–1.47	< 0.001
Quartile 3 (8.79–9.23)	1.28	1.19–1.38	< 0.001	1.86	1.76–1.96	< 0.001
Quartile 4 (> 9.23)	1.96	1.83–2.10	< 0.001	2.75	2.60–2.90	< 0.001
**Age ≥ 55 years**^b^						
**TyG index**	1.47	1.42–1.51	< 0.001	1.67	1.62–1.71	< 0.001
**TyG index**						
Low TyG index^c^	ref			ref		
High TyG index^c^	1.47	1.41–1.54	< 0.001	1.75	1.68–1.81	< 0.001
**TyG index (Quartiles)**						
Quartile 1 (< 8.37)	ref			ref		
Quartile 2 (8.37–8.78)	0.98	0.92–1.05	0.531	1.29	1.22–1.37	< 0.001
Quartile 3 (8.79–9.23)	1.11	1.04–1.19	0.001	1.72	1.63–1.82	< 0.001
Quartile 4 (> 9.23)	1.70	1.60–1.81	< 0.001	2.46	2.33–2.60	< 0.001

*AOR* adjusted odds ratio

^a^Adjusted for age, body mass index, region, scheme, year, smoking status, alcohol use, exercise, systolic blood pressure, diastolic blood pressure, serum uric acid

^b^Adjusted for sex, body mass index, region, scheme, year, smoking status, alcohol use, exercise, systolic blood pressure, diastolic blood pressure, serum uric acid

^c^ALT low TyG index is ≤ 8.84 and high TyG index is > 8.84, AST Low TyG index is ≤ 8.93 and high TyG index is > 8.93

^d^Low TyG index is ≤ 8.57 and high TyG index is > 8.57 for both AST and ALT

#### Receiver operating characteristics curve analysis of the TyG index prediction of elevated AST and ALT

Supplementary Fig. 1 illustrates the ability of the TyG index to predict elevated ALT. Supplementary Fig. 1a displays the overall AUC of 0.66 using the TyG index for predicting elevated ALT with an optimal cut point of 8.84 using Youden’s index (sensitivity 0.63, specificity 0.60). Supplementary Fig. 1b and c demonstrate the receiver operating characteristic (ROC) curves for males and females, respectively. The AUC was 0.68 using the TyG index for predicting elevated ALT in females with an optimal cut point of 8.57. Meanwhile, the optimal cutoff point for the TyG index for predicting elevated ALT in males was 8.89. Supplementary Fig. 1d, e, and f indicate the ROC curves for the 35–44, 45–54, and 55 and above age groups, respectively. The AUC using the TyG index for predicting high AST is shown in Supplementary Fig. 2.

## Discussion

In the present study, 232,235 RTA personnel aged 35–60 were enrolled nationwide. Previously, some studies in China demonstrated the association between the TyG index and liver function parameters among a healthy obese population [13, 14]. However, the current study is the most extensive study determining the relationship between the TyG index and aminotransferase among RTA personnel in Thailand. The dose‒response relationship between the TyG index and aminotransferase was demonstrated. A high TyG index was also strongly associated with elevated aminotransferases in both sexes and all age groups. The TyG index showed fair predictive ability when utilized for predicting aminotransferase. Furthermore, the prevalence of elevated aminotransferases in the population was investigated across sexes and age groups using TyG index quartiles.

The TyG index was found to reveal a dose‒response relationship with aminotransferases, particularly ALT. Similar to our study, a study in China identified a linear dose‒response relationship between the TyG index and ALT of 1.22 IU for every 1-unit increase in the TyG index [13]. The relationship may be explained as follows. The TyG index is known to be a simple novel indicator made up of TG and FPG, two laboratory tests that are key components of metabolic syndrome and cardiometabolic risk factors [9]. The TyG index is a marker for the severity of insulin resistance, which has been linked to the development and prognosis of metabolic diseases [9]. Elevated aminotransferases are two of the most common indicators used to test for hepatocellular injuries. AST is predominantly found in the heart, liver, skeletal muscle, and kidney, while ALT is predominantly found in the liver and kidney [20, 21]. ALT increases are thus more specific for liver damage [22]. Plasma aminotransferase levels are elevated due to a variety of medical conditions, the most prevalent of which is NAFLD [23].

Several cardiometabolic risk factors and insulin resistance were also found to be associated with aminotransferases [18–20]. Therefore, the independent association between insulin resistance and liver aminotransferase and the TyG index, known to be a good surrogate of insulin resistance, may explain the existence of a positive association between the TyG index and aminotransferase [9, 24, 25].

In the current study, TyG index thresholds of 8.89 and 8.57 for males and females, respectively, were developed to identify individuals with elevated ALT. The cut points and ability to predict elevated ALT were similar to those in another related Chinese study. However, their cut points for males were lower than those for females: 8.69 for males (sensitivity 70.2% and specificity 56.2%) and 8.96 for females (sensitivity 52.9% and specificity 70.1%) [26]. Their cut points among females might be higher since their definition of elevated serum ALT was above 40 U/L for both sexes. However, the cut points for females with elevated ALT in our case were 35 U/L. Another study from Bangladesh determined a similar cutoff value of 8.85 (sensitivity 93.5% and specificity 79% to predict NAFLD), and research from China found a similar cutoff value of 8.5 (sensitivity 72.2% and specificity 70.5%) [27, 28]. The present study indicated an association between a high TyG index and elevated aminotransferase.

Sex discrepancies were observed in the present study. A higher dose‒response relationship between the TyG index and ALT was observed among females than among males. The sex-related difference in fat distribution might explain the relationship. While females had higher total body fat compared to males, males had higher values for waist girth and visceral adipose tissue [29, 30]. Moreover, the prevalence of elevated aminotransferase was higher in males than in females. This may be due to the lower insulin resistance among females in comparison with males of similar age, particularly before menopause [31]. Furthermore, males are known to exhibit higher behavioral risks, including smoking and alcohol consumption, causing a higher prevalence of elevated aminotransferases in comparison with females [32, 33].

Differences in the relationship between the TyG index and aminotransferase were also observed between the different age groups. The prevalence of elevated ALT was shown to decrease in the higher age group. The reduction in prevalence in older age groups may be due to a reduction in ALT as we age [34]. A few published studies, such as ours, found that the prevalence of elevated ALT decreases with age [34]. This may be explained by the preserved regenerative response of the aged liver, a reduction in the stress protein response suggesting a reduction in homeostatic capacity, and a lower inflammatory response during aging [35]. However, the prevalence of glucose intolerance and hyperglycemia increases with age [36]. Consequently, a stronger dose‒response relationship between the TyG index and aminotransferase was observed in lower age groups in comparison with higher age groups, as well as a higher association between the TyG index and elevated aminotransferase.

Several cohort studies among the Chinese also indicated a strong association between the TyG index and NAFLD in those aged < 40 years, in both the diabetes and nondiabetes populations [37, 38]. In conclusion, those with a high TyG index, particularly those in the younger age group, may be a good population to screen for elevated aminotransferase. Moreover, NAFLD is a common cause of liver disease in adolescents and children. Thus, a high TyG index might be a good indicator for NAFLD and elevated aminotransferases in this population [39]. However, to the best of our knowledge, there are limited data on the relationship between the TyG index and NAFLD and elevated aminotransferase in these populations [40, 41].

Elevated aminotransferases are associated with NAFLD and insulin resistance [6, 42, 43]. Our study revealed a strong association between the TyG index and elevated aminotransferases, especially ALT. The lower age group and male population might require greater attention, as they were found to have a high prevalence of elevated aminotransferase, particularly those with a high TyG index. Lifestyle modification to reduce elevated aminotransferases and insulin resistance might be recommended, such as nutritional improvement, regular physical activity, weight loss, and alcohol restriction [44, 45]. Furthermore, even though NAFLD and elevated aminotransferases are mostly asymptomatic, undetected NAFLD may result in chronic liver disease and hepatocellular carcinoma [6]. Therefore, among those with a high TyG index, further screening of aminotransferase and ultrasonography might be encouraged for early detection of NAFLD and its severity [6, 46].

### Study strengths and limitations

The present study encompassed a substantial number of strengths, enrolling a large, representative sample of RTA personnel nationwide. The study provided valuable insights into the relationship between the TyG index and aminotransferase as well as the prevalence of elevated aminotransferase in each TyG index quartile in Thailand. Furthermore, future strategies for the primary prevention of NAFLD and elevated aminotransferases may benefit from these findings.

Our research encountered several limitations. First, the study employed a serial cross-sectional design in which the TyG index and aminotransferase were measured concurrently. Consequently, a causal relationship between the TyG index and elevated aminotransferase was not established. Second, the present study was conducted among RTA personnel, with a higher percentage of male participants (roughly 90%). Nevertheless, the results reflected an actual scenario in the study population. Third, due to the observational study using previously collected data from the health examination database, behavioral risk variables were collected very crudely. For instance, the total number of alcoholic drinks, the total number of cigarettes, and exercise intensity were not recorded. Finally, the data on infections such as viral hepatitis B and the drug usage of participants, which could alter the aminotransferase, were limited. Further adjustments for additional confounders and a meta-analysis are required to determine whether the TyG index can be utilized as a simple screening tool in medical practice.

## Conclusion

Our data revealed that elevated aminotransferases are a common health concern, particularly among males, participants aged 35–44, and those with a high TyG index. We also indicated the association between the TyG index and aminotransferase, showing that the TyG index is a novel risk factor for elevated aminotransferase in the RTA population.

## Supplementary Information

**Additional file 1: Figure S1.** ROC analysis of the triglyceride-glucose (TyG) index for predicting elevated alanine aminotransferase (ALT): a) overall; b) males; c) females; d) age group 35–44 years; e) age group 45–54 years; f) age group 55–60 years. **Figure S2.** Overall ROC analysis of the triglyceride-glucose (TyG) index for predicting elevated aspartate aminotransferase (AST).

## Data Availability

Because the data set contains identifying information, it cannot be shared publicly; additionally, the data belong to the Royal Thai Army Medical Department. Thus, ethical restrictions exist concerning the data set. Data are available from the Royal Thai Army Medical Department, Bangkok, Thailand, for researchers meeting the criteria to access confidential data.

## Abbreviations

NAFLD: Nonalcoholic fatty liver disease
TyG index: Triglyceride glucose index
AST: Aspartate aminotransferase
ALT: Alanine aminotransferase
FPG: Fasting plasma glucose
BMI: Body mass index
SBP: Systolic blood pressure
DBP: Diastolic blood pressure
HT: Hypertension
AOR: Adjusted odds ratio
CI: Confidence interval
SD: Standard deviation
IQR: Interquartile range
