# Supplementary material for: Relationship between triglyceride-glucose index and aminotransferase among Royal Thai Army personnel 2017–2021: a serial cross-sectional study
Source: Lipids Health Dis. 2023 Apr 3;22:47. doi: 10.1186/s12944-023-01811-5 (PMC10069073; doi:10.1186/s12944-023-01811-5)
Supplement: Supplementary file 1 — Additional file 1: Figure S1. ROC analysis of the triglyceride-glucose (TyG) index for predicting elevated alanine aminotransferase (ALT): a) overall; b) males; c) females; d) age group 35–44 years; e) age group 45–54 years; f) age group 55–60 years. Figure S2. Overall ROC analysis of the triglyceride-glucose (TyG) index for predicting elevated aspartate aminotransferase (AST). [file 12944_2023_1811_MOESM1_ESM.pdf]

Appendix

Relationship between Triglyceride-Glucose Index and Aminotransferase among Royal Thai Army Personnel 2017–2021: A Serial Cross-Sectional Study

Sethapong Lertsakulbunlue[1], Mathirut Mungthin[2], Ram Rangsin[3], Anupong Kantiwong[1] *Boonsub Sakboonyarat[3]

[1]Department of Pharmacology, Phramongkutklao College of Medicine, Bangkok 10400, Thailand
[2]Department of Parasitology, Phramongkutklao College of Medicine, Bangkok 10400, Thailand
[3]Department of Military and Community Medicine, Phramongkutklao College of Medicine, Bangkok 10400, Thailand

Table of contents

Figure S1. ROC analysis of the triglyceride-glucose (TyG) index for predicting elevated alanine aminotransferase (ALT): a) overall; b) males; c) females; d) age group 35–44 years; e) age group 45–54 years; f) age group 55–60 years. Page 3

Figure S2. Overall ROC analysis of the triglyceride-glucose (TyG) index for predicting elevated aspartate aminotransferase (AST). Page 4

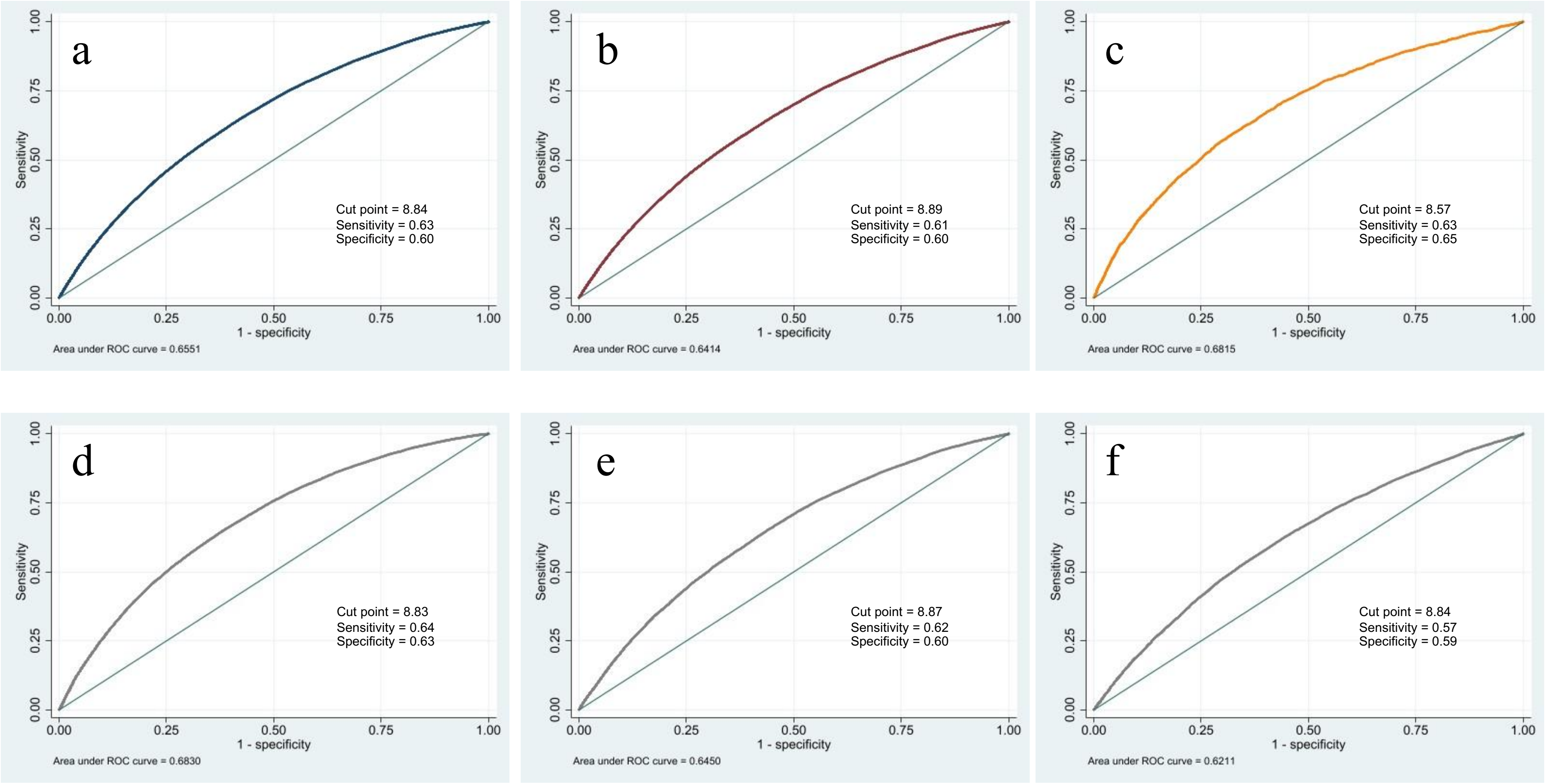

Figure S1. ROC analysis of the triglyceride-glucose (TyG) index for predicting elevated alanine aminotransferase (ALT): a) overall; b) males; c) females; d) age group 35–44 years; e) age group 45–54 years; f) age group 55–60 years.

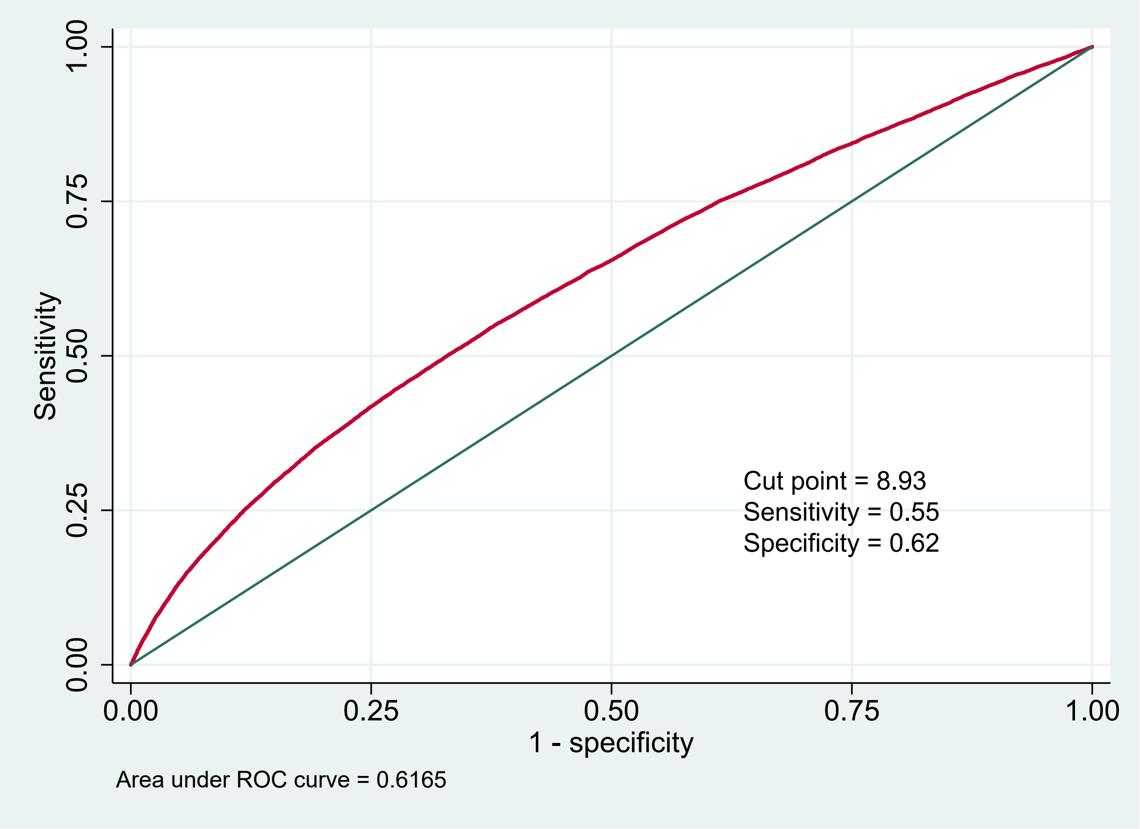

Figure S2. Overall ROC analysis of the triglyceride-glucose (TyG) index for predicting elevated aspartate aminotransferase (AST).
